# Metal Contents of Lake Fish in Area Close to Disposal of Industrial Waste

**DOI:** 10.1155/2021/6675374

**Published:** 2021-04-21

**Authors:** Suyud Warno Utomo, Frisca Rahmadina, Bambang Wispriyono, Haryoto Kusnoputranto, Al Asyary

**Affiliations:** ^1^Department of Environmental Health, Faculty of Public Health, Universitas Indonesia, Depok, Indonesia; ^2^Study Program of Environmental Science, School of Environmental Sciences, Universitas Indonesia, Depok, Indonesia

## Abstract

This research was conducted to analyze the content of Fe, Cu, Cd, Cr, and Pb in several species of fish taken from three lakes that are close to the disposal of industrial waste in Indonesia. The fish samples were taken from three lakes, namely, Muara Angke, Weda, and Morowali. The samples from Morowali were analyzed in April 2019, those from Weda from November to December 2019, and those from Muara Angke in June 2018. All the samples were then analyzed at the Chemistry Laboratory of the Department of Chemistry, Faculty of Mathematics and Natural Science, University of Indonesia, and the Integrated Laboratory of IPB. The main results showed that all types of fish from Morowali and Weda were no longer safe to consume because they contained Fe, Cu, Cd, and Cr exceeding the threshold of metal contamination. Meanwhile, all types of fish from Muara Angke, except for *ayam-ayam*, are still safe for consumption. The results of this study can be a source of information regarding metal content in fish and fish feed for safe consumption. Given the high consumption rate of fish and the hazards of heavy metals on humans' health, such research must be furthered.

## 1. Introduction

Every activity will always have an impact on the environment. The term impact itself varies from country to country; meanwhile, in Indonesia, according to the laws and regulations, it is interpreted as change. Change can be a positive direction which means a positive impact but can also be a negative direction which means a negative impact. The philosophy of sustainable development is to minimize negative impacts and maximize positive impacts. The size of the activity and the complexity of the activity in the location of the activity will be highly correlated with the impact it will cause. Various activities that can lead to changes in landscape, changes in land use, and changes in land ownership and activities that have the potential to cause waste and environmental pollution must be anticipated from the beginning so that impact development can be continued.

Mining is an industry where in its activities it involves extracting mineral materials from the earth [1]. Indonesia has enormous opportunities in mining because it is supported by the vast territory of Indonesia which is rich in mineral resources. The mining industry in Indonesia is carried out by domestic companies (state-owned and private) and multinational companies [2]. Mining has three important components in its activities, including exploration, mining, and processing. In the early stages of mining, the proponent must have information about the location of the mine and estimate the value of the mineral ore deposit that will be obtained during the exploration phase. If the mineral ore exploration phase proves there is a large enough mineral ore, the initiator will start the mine development process [1]. The process used by the mining industry to obtain economical minerals can use the extraction method. The extraction method is the process of separating the minerals from the rock to the following minerals which are no longer needed. Unnecessary minerals will become waste in the mining industry and can cause environmental pollution (Ostensson & Roe 2017).

The development of industrial estates in the long term and the growing number of industrial sectors in the community, if not controlled, will have an impact on the regional structure and environmental pollution such as water pollution [3]. The presence of heavy metals in the water can accumulate in the bodies of organisms such as fish after being absorbed by the gills [4]. Heavy metals that enter a fish's body cannot be removed from it because of such accumulation. As a result, these heavy metals will continue to exist along the food chain. In addition, this accumulation can ultimately damage the fish's organs, resulting in its death. If the fish is then consumed by humans, this can cause chronic and acute poisoning. For example, the overload of Fe content can cause poisoning (vomiting), intestinal damage, impaired absorption of vitamins and minerals, and homochromia [5]. Se and other heavy metals such as lead can interfere with oxidase production and consequently hamper cell metabolism, affecting growth [6].

Several studies have been done to determine the metal pollutants contained by fish as it is a common meal consumed by humans [7]. Given the high consumption rate of fish and the hazards of heavy metals on humans' health, such research must be furthered. This study aimed at analyzing the content of metals in fish taken from lakes that are close to the disposal of industrial waste in Indonesia.

## 2. Materials and Methods

### 2.1. Study Setting

The fish samples were taken from three lakes, namely, Muara Angke, Morowali, and Weda. The first five samples were taken from Morowali in April 2019, namely, the layur, sarong, tracan, taking, and krau fish species. The subsequent fish samples were taken from Weda in November 2019, namely, yellow selar, flying fish, oci, and yellow fish. Meanwhile, at Muara Angke, the fish samples consisted of ayam-ayam, black pomfret, red snapper, kuwe, mackerel, and mackerel tuna.

### 2.2. Data Collection

The fish samples from Morowali were analyzed in April 2019 at the Chemistry Laboratory of the Department of Chemistry, Faculty of Mathematics and Natural Science, University of Indonesia; the fish samples from Weda were analyzed from November to December 2019 in the same facility. Meanwhile, the fish samples from Muara Angke were analyzed in June 2018 at the Integrated Laboratory of IPB.

### 2.3. Instrument and Data Analysis

The tools used in this study were clear plastic bags, cool boxes, label paper, atomic absorption spectrophotometer cameras, ovens, digital balances, hotplates, filter paper, Petri dishes, porcelain mortars, desiccators, analytical scales, and common laboratory glassware. Data obtained from the measurements were analyzed by descriptive comparison with raw data of the National Standardization Agency, which set the limits of metal contaminant weight in fish meals.

Univariate analysis was performed by the data distribution for each variable, respectively. This presentation analysis was resulting the value that can represent the quality of the environment.

### 2.4. Details of Experiment

**Figure 1:**
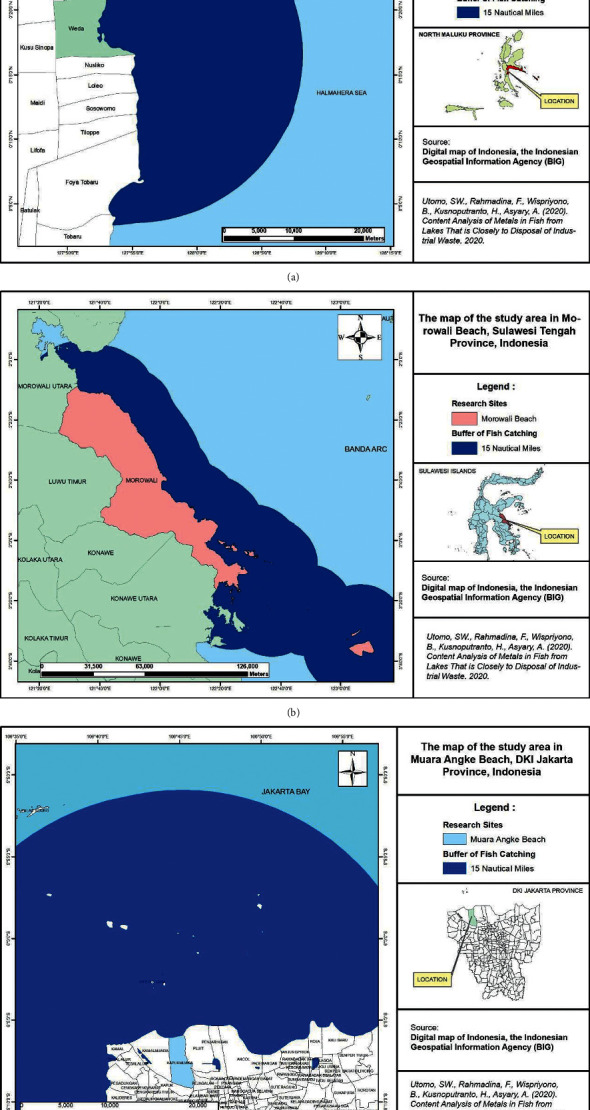
The map of the study area: (a) Weda; (b) Morowali; (c) Muara Angke.

Sampling method: there were three kinds of fishes that were selected by both most captured by fisherman and most consumed by residents. The sampling technique was carried out 15 miles from the lakeside, by distributing three points in each 5 miles (Figures 1(a)–1(c)). Number of samples: each of the three fishes was represented in 30 samples. This sample size was calculated according to the standard sample minimum of health environmental risk assessment. Sample preparation: (1) Fishes were washed and weighted (kg), and measured (cm). (2) The body of fishes was taken from its bones. (3) Fishes were mashed and weighted (0.5 g). (4) 5 ml of 65% HNO_3_ was added to the specimens and the specimens were left for 15 minutes in the acid room, and this phase is to dissolve the organic compound in the specimens. (5) Specimens were kept in a microwave oven at a temperature of 200°C for 20 minutes. The advantage of this phase is no volatile elements vanished and the needed time is relatively not too long. (6) After the specimen was cooled, it was washed with distilled water and transferred to a volumetric flask. (7) From the volumetric flask, the specimen was put into a test tube and centrifuged for 10 minutes at 150 rpm. The purpose of this step is to separate the suspended molecule. (8) Lastly, the specimen was put into an atomic absorption spectrophotometer (AAS) in order to observe its concentration visually.

### 2.5. Health Risk Assessment: Pb in Muara Angke

In this site, risk quotient (RQ) analysis was employed to measure health risk assessment by consumption of studied fish. 96 fishermen, who were ≥17 years old and had been domiciled at least one year in surrounding lakes, were selected according to the minimum sample size calculation [8].

Individual characteristics of respondents including body weight (Wb) and consumption rate (*R*) were measured, and duration (Dt) and frequency of exposure (fE) were also measured. These criteria were used to measure the intake of respondents (CDI). RQ was obtained by comparing CDI with reference dose (RfD). RfD was set according to the integrated risk information system (IRIS) as an international standard for Pb (4 × 10^−3^ mg/kg/daily) [9]. RQ assumes existed and it needs to manage when RQ value >1 [10].

## 3. Results and Discussions

The highest Fe content was found in taking (69.60 mg/kg). The highest Cu content was found in sarong (0.18 mg/kg). The highest Cd and Cr contents were found in krau (2.30 mg/kg and 8.41 mg/kg, respectively). No amount of lead was contained in all fish samples from Morowali (Table 1).

The highest Fe content was found in yellow selar (24.15 mg/kg). The highest Cu content was found in yellow selar 3 (0.48 mg/kg). No traces of Cd, Cr, and Pb were contained in all fish samples from Weda (Table 2).

The fish sample containing the highest Cd and Pb contents was ayam-ayam (0.0345 mg/kg and 0.428 mg/kg, respectively) (Table 3).

The heavy metal content in a fish's body is closely related to the disposal of industrial waste in and around the fish's habitat, such as rivers, lakes, and seas [11]. The accumulation of heavy metals in fish is caused by contact between the fish and the aquatic medium containing heavy metals. Such accumulation can occur in several ways, such as in the respiratory and food channels and through the skin [12]. The metals are absorbed into the flesh of the fish by its blood, bound to the blood's proteins, and then distributed to all the body tissues [13]. The highest metal accumulation is usually found in the liver and kidneys. The accumulation of heavy metals in body tissues occurs first in the gills, the liver, and then the meat of the fish [14]. The amount of heavy metals absorbed by and distributed in the fish depends on the form of compounds and concentrations of the pollutants, the activity of the microorganisms, the sediment texture, and the types of fish that live in the neighborhood.

### 3.1. Metal Content in Morowali Fish

The metal content in the fish samples from Morowali, from the highest to the lowest levels, was as follows: Fe, Cr, Cd, Cu, and Pb. When compared with data from the National Standardization Agency (2009) regarding Fe contamination in food, all the fish samples from Morowali exceeded the limit, which is 1 mg/kg. The Cr content in the fish samples was compared with data from the Director General of POM Decree No. 03725/B/SK/89 concerning Cr contamination in food; all the fish samples exceeded the threshold, which is 2.5 mg/kg [15].

The Cd content in layur and krau exceeded the limit of Cd contamination in food, which is 0.2 mg/kg [15]. The Cu content in sarong exceeded the threshold of Cu allowed in food, which is 0.02 mg/kg. The Pb content in all the Morowali fish samples was still below the threshold, which is 0.3 mg/kg [15]. To sum, the fish samples from Morowali that exceeded the threshold of metal contamination are no longer safe for consumption.

### 3.2. Metal Content in Weda Fish

The fish samples derived from Weda only contained Fe and Cu. When compared with data from the National Standardization Agency (2009) regarding the limit of Fe contamination in food, all the fish samples from Morowali exceeded the limit, which is 1 mg/kg. The Cu content in the fish samples was also compared with data from the National Standardization Agency (2009) regarding the limit of Cu contamination in food. The yellow selar (2 and 3) and oci exceeded the specified threshold, which is 0.02 mg/kg. Meanwhile, the results of the analysis did not show the presence of Cr, Cd, and Pb in all the fish samples from Weda. To sum, the fish samples from Weda that exceeded the threshold of metal contamination are no longer safe for consumption.

### 3.3. Metal Content in Muara Angke Fish

When compared with data from the National Standardization Agency (2009) regarding the limit of Pb contamination in food, only the ayam-ayam sample exceeded the threshold, which is 0.3 mg/kg. No level of Cd content in the samples exceeded the limit of Cd contamination in food. Thus, only the ayam-ayam is not safe for consumption.

The high concentration of Fe in all the samples from Morowali and Weda can be caused by several sources (i.e., apart from the land, human activities that occur on the mainland), for example, the discharge of domestic sewage containing iron, the water reservoir containing iron, industrial waste deposits, and the corrosion of water pipes containing ferrous metals leading to the ocean. Increasing concentrations of iron can also be caused by the erosion of mineral rocks from pounding waves and wind as well as the corrosion of rusting ships and ports [16].

Fe has an important role in the process of enzymatic oxidation, cytochromes, and respiratory pigments (hemoglobin), but the excess of Fe can lead to poisoning. It can cause vomiting, impaired intestines, premature aging to sudden death, irritability, arthritis, birth defects, bleeding gums, cancer, cirrhosis of the kidneys, constipation, diabetes, diarrhea, dizziness, fatigue, blackish skin, headaches, liver pain and failure, hepatitis, hyperactivity, infections, insomnia, mental health problems, metallic taste in the mouth, rheumatism, cytopenia, the impaired absorption of vitamins and minerals, and homochromia [17].

The high level of Cr contained in fish from Morowali can be caused by activities in the textile (e.g., fabric coloring), paint, leather tanning, metal coating, or battery industries. Cr infestation into the food chain deposited in fish can cause poisoning. Cr is an important nutrient in carbohydrate metabolism, but at higher concentrations, it can be toxic [18]. It has a negative impact on the liver, kidneys, and protoplasm in cells, producing carcinogens (cause cancer), teratogens (inhibit fetal growth), and mutagens [19].

In addition, the presence of Cu in fish from Morowali and Weda can be derived from antifouling, widely used for fishing boats. Antifouling is used to seal the vessel and reduce damage to the boat by organisms. The existence of fish in poor environmental conditions can also cause the fish to accumulate heavy metals. Such a situation occurs because fishing locations are correlated with industrial waste and the transportation hubs of ships. The shipping industry and transportation are major sources of heavy metal pollution in waters.

Cd contamination in fish from Morowali and Muara Angke indicates the presence of Cd pollutants in these lakes. Cd contamination can be due to waste disposal by industries that use Cd for electric and galvanic coating processes as Cd is noncorrosive. Cd metal poisoning can be characterized by symptoms of back pain for several years and eventually osteomalacia or the softening of bone and spinal fractures.

The content of Pb in all samples from Muara Angke indicates Pb pollution caused by the contamination of fuel ships. Based on the analysis of Pb content in fish samples in Muara Angke, only ayam-ayam exceeded the limit of Pb contamination, while the other fish samples are still safe for consumption. However, overexposure to Pb can disrupt the hemopoietic, nervous, kidney, gastrointestinal, cardiovascular, and reproductive systems in the human body.

### 3.4. Health Risk Assessment by Consumption of Studied Fish

RQ is usually used to estimate the risk that can be impacting human health as a result of exposure. The RQ value represents both the noncarcinogenic and excess cancer risk (ECR) of carcinogenic effects [20]. The risk quotient for noncarcinogenic effects can be calculated using the following formula:

(1) $$\begin{array}{c} \text{RQ}=\frac{\text{CDI}}{\text{RfD}}. \end{array}$$

CDI can be calculated by

(2) $$\begin{array}{c} \text{CDI}=\;\frac{C\times R\times \text{fE}\times \text{Dt}}{\text{Wb}\times \text{tavg}}, \end{array}$$

where CDI is the intake (0.4 mg/kg/daily), *C* is the concentration of Pb (0.4 mg/kg of ayam-ayam fish), *R* is the consumption rate of fish (0.6900 kg/daily), fE is the frequency of exposure (64 days/year), Dt is the duration of exposure (15 years), Wb is the weight of the body (58.300 kg), and tavg is the time of period (30 years × 365 days).

(3) $$\begin{array}{c} \text{CDI}=\frac{0.4\text{mg}/\text{kg}\times 0.6900\text{kg}/\text{daily}\times 64\text{days}/\text{year}\times 15\text{ years}}{58.300\text{ kg}\times 30\text{ years}\times 365\text{ days}},\\ \text{CDI}=0.00041505\frac{\text{mg}/\text{kg}}{\text{daily}}. \end{array}$$

With RfD = 4 × 10^−3^ mg/kg/daily, thus

(4) $$\begin{array}{c} \text{RQ}=\frac{0.00041505\left(\text{mg}|/|\text{kg}\right)/\text{daily}}{0.004\left(\text{mg}|/|\text{kg}\right)/\text{daily}},\\ \text{RQ}=0.103762. \end{array}$$

**Figure 2:**
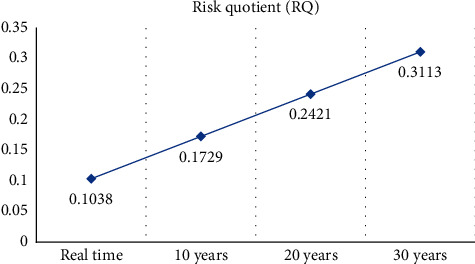
Risk quotient (RQ) in prospective duration estimation.

According to the RQ result <1, it can be concluded that it is still quite safe to consume the ayam-ayam fish at the moment. In addition, if we calculate the duration of exposure for the long term, e.g., 10, 20, and 30 years further, RQ is still below 1 (Table 4). It means that this kind of fish would be relatively safe to consume even after 30 years further (Figure 2).

By tailoring study findings, contribution of metals as the pollutant can be estimated. The ecological risk posed by the surface of the lake can be managed by mitigating sustainable development which concerned the healthy environment [20]. In addition, law enforcement according to regional regulation is also needed [21], and this action would be powerful to optimize the harm of industrial waste in the environment.

## 4. Conclusions

In conclusion, all types of fish from Morowali are no longer safe for consumption because they contain traces of Fe, Cu, Cd, and Cr that exceed the thresholds of metal contamination. All types of fish from Weda are no longer safe for consumption as well because they contain levels of Fe and Cu that exceed their respective thresholds. Meanwhile, all types of fish from Muara Angke, except *ayam-ayam*, are safe for consumption.

## Figures and Tables

**Table 1:** Content of Fe, Cu, Cd, Cr, and Pb in fish samples from Morowali.

Test sample	Fe (mg/kg)	Cu (mg/kg)	Cd (mg/kg)	Cr (mg/kg)	Pb (mg/kg)
Layur	25.11	ND	1.93	3.37	ND
Sarong	8.77	0.18	ND	4.42	ND
Tracan	42.25	0.06	ND	5.28	ND
Taking	69.60	ND	ND	7.72	ND
Krau	65.42	ND	2.30	8.41	ND

ND: not detected.

**Table 2:** Content of Fe, Cu, Cd, Cr, and Pb in fish samples from Weda.

Test sample	Fe (mg/kg)	Cu (mg/kg)	Cd (mg/kg)	Cr (mg/kg)	Pb (mg/kg)
Yellow selar (Weda)	24.15	ND	ND	ND	ND
Yellow selar 2 (Weda)	21.16	0.47	ND	ND	ND
Yellow selar 3 (Weda	20.54	0.48	ND	ND	ND
Flying fish (Sagen)	7.24	ND	ND	ND	ND
Oci (Sagen)	15.12	0.13	ND	ND	ND
Small yellow selar (Lelilef)	4.00	ND	ND	ND	ND
Large yellow selar (Lelilef)	4.97	ND	ND	ND	ND

ND: not detected.

**Table 3:** Content of Cd and Pb in fish samples from Muara Angke.

Test sample	Cd (mg/kg)	Pb (mg/kg)
Ayam-ayam	0.0345	0.428
Black pomfret	0.0210	0.228
Red snapper	ND	0.106
Kuwe	0.0005	0.190
Mackerel	ND	0.206
Mackerel tuna	0.0005	0.142

ND: not detected.

**Table 4:** Risk quotient (RQ) in prospective duration estimation.

Health risk parameters		Ayam-ayam fish
CDI (mg/kg/daily)	Real time	0.000415
	10 years	0.000692
	20 years	0.000968
	30 years	0.001245
RfD		0.004
RQ	Real time	0.1038
	10 years	0.1729
	20 years	0.2421
	30 years	0.3113

## Data Availability

The data used to support the findings of this study are available from the corresponding author on request. This article's content was previously made public on a preprint server https://www.researchgate.net/publication/344531734_Content_Analysis_of_Metals_in_Fish_from_Waters_That_are_Closely_to_Disposal_of_Industrial_Waste.
